# Geographic accessibility to addiction, mental health, and HIV/AIDS health-care services for opioid-dependent clients

**DOI:** 10.1186/1940-0640-10-S1-A10

**Published:** 2015-02-20

**Authors:** Curtis J Denton, Debarchana Ghosh, Faye S Taxman, Frederick L Altice

**Affiliations:** 1 Department of Geography, University of Connecticut, Storrs, CT, 06269, USA; 2 Criminology, Law and Society Department, George Mason University, Fairfax, VA, 22030, USA; 3 Yale School of Medicine, Yale University, New Haven, CT, 06520, USA

## Background

Access to care is an important concept in health policy and health services research, yet it is one which has not been defined precisely. To some researchers “access” refers to entry into or use of a health-care system, while to others it characterizes factors influencing entry or use. [1] This paper studied accessibility as it refers to entry into or use of a health-care system by measuring geographic accessibility from the location of clients (opioid-dependent individuals) to preventive and treatment facilities for substance use disorders (SUDs), mental health, and HIV/AIDS.

## Materials and methods

The study population is opioid-dependent clients (N = 530) screened for an ongoing study of opioid substitution therapy (OST) in Washington, DC. There were three major methodological steps. First, the study geocoded and mapped the spatial distribution of clients’ self-reported neighborhood of residence (algorithmically moved to a nearby location to protect privacy) and health-care facilities specific to their addiction and comorbidities. The facilities included centers for prevention, OST (buprenorphine and methadone), and recovery from SUDs, mental health, STI clinics and HIV/AIDS, harm reduction sites (needle exchanges), and community support (counseling centers, AA, and NA meetings). Second, the study used geostatistical methods to measure accessibility (distance and travel time) from the location of clients to the health-care facilities. Third, the study constructed Addiction Severity Index scores using client questionnaires to measure correlations between opioid users and treatment services.

## Results

**Figure 1:**
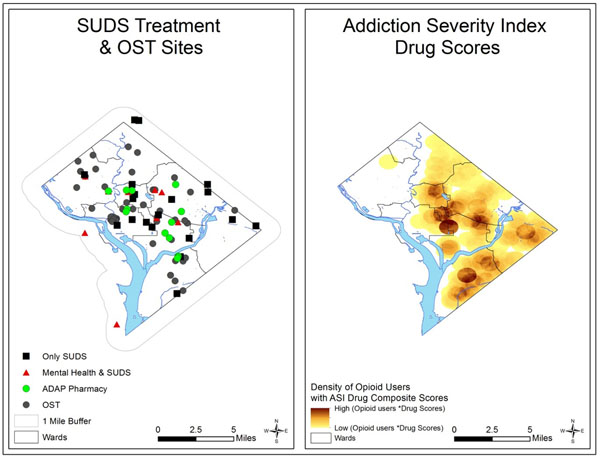


The majority of the opioid-dependent clients were located in the south-central and southern parts of the District, with significantly higher concentration in the southeast. On the other hand, the preventive and treatment centers for SUDs and mental health issues were concentrated in the south-central region with fewer substance abuse services, OST providers (Figure 1), mental health services, and AIDS drug assistance program-affiliated pharmacies in the southeast. Furthermore, there was only one site for needle exchange in this area. The results indicate that health care services needed by opioid-dependent clients with their comorbidities are not geographically accessible to those clients.

## Conclusions

Often times, it is the individuals with the greatest need for addiction and related health-care services who are the ones with the least geographic accessibility to those services. These findings highlight the need for more careful consideration of geographic accessibility and neighborhood-level contextual barriers in evaluating and planning access to addiction and related health-care services.

## References

[B1] Penchansky R Thomas W The concept of access: Definition and relationship to consumer satisfaction Medical Care 1981 19 2 10.1097/00005650-198102000-00001 7206846

